# Primary data, claims data, and linked data in observational research: the case of COPD in Germany

**DOI:** 10.1186/s12931-018-0865-1

**Published:** 2018-08-30

**Authors:** Sabrina Mueller, Fraence Gottschalk, Antje Groth, Wilhelmine Meeraus, Maurice Driessen, Thomas Kohlmann, Thomas Wilke

**Affiliations:** 1 IPAM e.V., Alter Holzhafen 19, 23966 Wismar, Germany; 2 GSK, 980 Great West Road, Brentford, Middlesex, TW8 9GS UK; 3 Universitätsmedizin Greifswald, Institut für Community Medicine, Walther-Rathenau-Str. 48, 17475 Greifswald, Germany; 4 Ingress-Health, Alter Holzhafen 19, 23966 Wismar, Germany

**Keywords:** Observational research, Claims data analysis, Chronic obstructive pulmonary disease, Real-world evidence study, Linked data study

## Abstract

**Background:**

Real-world evidence (RWE) can inform patient management decisions, but RWE studies are associated with limitations. Linkage of different RWE data types could address such limitations by enriching data and improving scientific quality. Using the example of chronic obstructive pulmonary disease (COPD) in Germany, this study assessed the value of data linkage between primary and secondary data sources for RWE.

**Methods:**

Post hoc analysis of data from an observational RWE study, which used prospectively collected data and data from an insurance claims database to assess treatment adherence and persistence in patients with COPD in Germany. Patient-level primary data were collected from the prospective observational study (primary dataset, *N* = 636), and claims data from the sickness fund AOK Nordost (claims dataset, *N* = 74,916). Primary and claims data were linked at a patient level using insurance numbers (linked dataset). Patients in the linked dataset were indexed at date of study inclusion for primary data and matched calendar date for claims data. Agreement between primary and claims data was examined for patients in the linked dataset based on comparisons between recorded sociodemographic data at index, comorbidities (primary: any recorded; claims: pre-index), prescriptions for COPD therapies (type and date) and exacerbations in the 12-month post-index period.

**Results:**

The linked dataset included primary and claims data for 536 patients. Fewer comorbid patients were reported in primary data compared with claims data (*p* < 0.001), with overall agreement between 63.6% (hypertension) and 90.5% (osteoporosis). Number of prescriptions for COPD therapies per patient was lower in primary versus claims data (3.7 vs 10.3 prescriptions, respectively), with only 24.5% of prescriptions recorded in both datasets. Only 11.5% of exacerbations (moderate or severe) were recorded in both datasets, with 15.5% recorded only in primary data and 73.0% recorded only in claims data.

**Conclusion:**

Our study highlighted discrepancies between primary and claims data capture for this population of German patients with COPD, with lower reporting of comorbidities, COPD therapy prescriptions and exacerbations in primary versus claims data. Study findings suggest that data linkage of primary and claims data could provide enrichment and be useful in fully describing COPD endpoints.

**Electronic supplementary material:**

The online version of this article (10.1186/s12931-018-0865-1) contains supplementary material, which is available to authorized users.

## Background

Analyses of real-world evidence (RWE) are becoming increasingly popular in all therapy areas, including respiratory diseases [1, 2]. This is largely because data regarding the effectiveness and safety of treatments are critical to guide treatment decisions by physicians and decision makers/payors, as they are more generalizable to daily care than data from randomized clinical trials with strict inclusion/exclusion criteria [2, 3]. However, issues concerning the appropriateness of RWE collection methods, analysis methods and data reliability have been previously highlighted [4, 5].

Many study designs and data sources are available for RWE generation, which mainly involve primary data collection or secondary data use [5, 6]. Primary data studies document data from patients explicitly included for study purposes, and may collect data either retrospectively, via patient charts/other data sources, or prospectively, via documentation of data by study physicians/patients. Secondary data studies use data that have been previously recorded for reasons other than the intended study objectives in a retrospective manner. Such data may, for example, be obtained from administrative claims databases, existing patient registries, or electronic medical record databases [7].

Most RWE studies are based on a single data type; however, each data type is associated with its own strengths and weaknesses [6] and the appropriate choice of data type and study design is dependent on the scientific question asked. In addition, practical factors such as data availability, cost, generalizability of the data, project timelines and necessary approval processes including ethical approval may impact the choice of study design and data source [8, 9]. Data enrichment via linkage of different types of RWE could address some of the weaknesses associated with single source data capture and improve the scientific quality of the studies; however, data linkage may also be associated with limitations such as selection bias (e.g. when linking study populations with different inclusion/exclusion criteria), potential linking errors [10], or a potential loss of power due to smaller sample size.

To gain insight into the value of linking primary and secondary data sources, this analysis assessed the degree of agreement between such data sources using the example of chronic obstructive pulmonary disease (COPD) in Germany.

## Methods

### Study design and objectives

This was a post hoc analysis of data from an observational study aiming to assess treatment adherence and persistence in patients with COPD in Germany (GSK study HO-12-8607). Adherence and persistence were assessed in two ways: using data purposely collected from physicians and patients at multiple centers (via retrospective chart review and prospective data collection), and using insurance claims data from the AOK Nordost database (German sickness fund which insures patients located in the regions of Berlin, Brandenburg and Mecklenburg Western Pomerania). The observational study was approved by the ethics committee of the University of Rostock.

The analysis presented here was conducted in three parts; (1) patient-level primary data were collected from the observational study (primary dataset), (2) patient-level data were also obtained from the retrospective cohort analysis (claims dataset), and (3) data from the primary dataset were linked to those from the claims dataset at a patient level (linked dataset).

The objectives of the analysis were to use the linked dataset to evaluate agreement between the primary dataset and the claims dataset, based on comparisons between sociodemographic data, comorbidities, prescriptions for COPD therapies and exacerbation events.

### Data capture

#### Primary dataset

Invitations to participate in the observational study were sent out randomly based on a known list of treating physicians (general practitioners [GPs] as well as independent outpatient pneumologists) in the North-East of Germany. Physicians were asked to include patients based on the following inclusion criteria: ≥40 years of age; Global initiative for chronic Obstructive Lung Disease (GOLD) stage 2–4 (moderate to very severe COPD; based on physician’s assessment) [11] or known to have had at least one exacerbation prior to the index date; currently on COPD maintenance therapy; and insured by the statutory health insurance fund AOK Nordost. The restriction of patients insured by a specific sickness fund was necessary to facilitate later linkage of primary data with claims data from those patients.

Enrollment of the first patient took place in March 2013. The last patient completed the study in August 2015. Patients provided written informed consent at enrollment (index date) and physicians were asked to document sociodemographic and clinical data in an electronic case report form for a 24-month pre-index period and a 12-month post-index observational period with three prospective documentations (at index, 6 months and 12 months; Additional file 1: Table S1 (see Supplementary Materials). While the observational study also collected patient-reported data (Additional file 1), only physician-reported data were considered in this analysis.

#### Claims dataset

Retrospective claims data was provided by the sickness fund AOK Nordost. Key inclusion criteria for the claims dataset were as follows: at least two outpatient diagnoses or one inpatient diagnosis of COPD (International Classification of Diseases, 10th revision [ICD-10] code J44*) between January 1, 2010 and December 31, 2015; and ≥ 40 years of age at first COPD diagnosis. Patients with a confirmed asthma diagnosis (ICD-10 code J45), or who could not be observed for at least 12 months after first COPD diagnosis (death not taken into account), were excluded. Data captured included sociodemographic characteristics, information regarding inpatient and outpatient care (including diagnoses and procedures) and outpatient prescriptions.

#### Linked dataset

Data linkage of primary and claims data was performed by a third-party clearing center (University of Greifswald, Germany) and undertaken at a patient level on the basis of insurance numbers, as well as a comparison of the name documented in the primary dataset (according to the informed consent form) and the claims dataset. Patients provided written informed consent for inclusion in the linked dataset. Following completion of the data linkage procedure, an anonymous linked dataset that contained all collected patient-level primary and claims data was created. The linked dataset index date was the primary dataset index date for primary data; matched calendar date for claims data.

### Evaluation of study objectives

To evaluate whether data available in the primary and claims datasets differed from each other this analysis compared (1) patient characteristics (sociodemographic data and comorbidities); (2) prescriptions of COPD therapies; and (3) COPD exacerbations.

1. Sociodemographic data (i.e. patient’s age and gender) were evaluated on the linked dataset index date. In the primary dataset, physicians were asked to document sociodemographic data at index. In the claims dataset, patient’s age at the linked dataset index date was calculated based on the recorded year of birth. Therefore, a difference of ±1 year in age between the datasets was treated as equal. Furthermore, for the primary dataset, study physicians were asked to document at index the existence of any comorbidities that they were aware of for each patient. These data were compared with documented diagnoses (based on ICD-10 codes) in the claims dataset baseline period (from January 1, 2010 to the linked dataset index date). For the claims dataset, a comorbidity was assumed to exist if at least one outpatient or one inpatient diagnosis of the respective disease was documented. For each comorbidity, the agreement between the primary and claims datasets was matched at a patient level. The overall degree of agreement for each comorbidity was calculated as follows:

$$ \% of\ agreement={100}^{\ast}\frac{\begin{array}{l}N\  of\ patients\ with\ comorbidity\ X\  in\ primary\ and\ claims\ data+\\ {}N\  of\ patients\ with out\ comorbidity\ X\  in\ primary\ and\ claims\ data\end{array}}{Total\ N\  of\ patients\ in\ the\ linked\ dataset} $$

This analysis was performed for all patients and separately for patients who had been included either by pneumologists or GPs as study physicians in the primary dataset. A sensitivity analysis was also performed, which considered comorbidities in the claims dataset as defined by at least two outpatient diagnoses or at least one inpatient diagnosis. An additional sensitivity analysis evaluated comorbidities reported in the claims dataset by considering only those comorbidities associated with prescription(s) for comorbidity-related medication(s).

(2). Prescriptions for COPD therapies (Additional file 1: Table S2 ) were evaluated during the 12-month post-index period (for primary data) and the matched calendar period (for claims data). Quality of documentation was defined based on: (i) prescriptions documented in both datasets (same medication and same prescription date in both datasets); (ii) prescriptions only documented in the primary dataset; and (iii) prescriptions only documented in the claims dataset. The degree of agreement between prescriptions documented for patients in the linked dataset was first calculated at a prescription level as follows:

$$ \% of\ agreement\ of\ prescriptions={100}^{\ast}\frac{N\  of\ prescriptions\ in\ primary\ and\ claims\ data}{Total\ N\  of\ prescriptions\ in\ the\ linked\ dataset} $$

This methodology was applied for each patient, to determine how many of their prescriptions in the linked dataset were documented in primary and claims data (same medication prescribed on the same day). Moreover, agreement was evaluated for the overall patient population in the linked dataset as well as separately for those patients who were included by either a GP or a pneumologist in the primary dataset. A sensitivity analysis was also performed, which only considered prescriptions for COPD maintenance therapies, i.e. long-acting muscarinic antagonists (LAMAs), long-acting β_2_-agonists (LABAs), inhaled corticosteroids (ICS), phosphodiesterase type 4 (PDE-4) inhibitors and methylxanthines. Agreement was further assessed at a patient level by evaluating, for each patient in the linked dataset, how many prescriptions for COPD therapies were recorded in primary data, claims data or both.

(3). The number of documented acute moderate and severe COPD exacerbations was evaluated for the 12-month post-index period (for primary data) and the matched calendar period (for claims data). For the primary dataset, physicians were asked to document any exacerbation requiring medical treatment (prescriptions for antibiotics or glucocorticoids) or hospitalization that occurred during the study period as far as they knew. In the claims dataset, outpatient and inpatient exacerbations (ICD-10 code J44.1 – acute exacerbation) were captured. Severe exacerbations were defined as those requiring hospitalization in the primary dataset, and as hospitalizations associated with an exacerbation ICD-10 code in the claims dataset. The degree of agreement between the two datasets was calculated at an exacerbation level (matched by patient and date) as follows:

$$ \% of\ agreement\ of\ exacerbations=\frac{100^{\ast }N\  of\ exacerbations\ in\ primary\ and\ claims\ data}{Total\ N\  of\ exacerbations\ in\ the\ linked\ dataset} $$

Agreement was evaluated for all patients in the linked dataset and separately for patients who were included by either a GP or a pneumologist in the primary dataset.

### Statistical analysis

Data were analyzed descriptively. Percentages, mean (standard deviations [SD]) and median (interquartile range [IQR]), and *p*-values for the comparisons between datasets, were provided where applicable. Statistical comparisons were performed using Pearson’s chi-squared test, Mann–Whitney U test or *t*-test, depending on the type and distribution of the variable. Significance was defined as *p* < 0.05. The Cohen’s kappa coefficient was used to determine the agreement between the values of comorbidity variables recorded in primary and claims data. The kappa coefficient was not used for exacerbations and prescriptions as data relating to these outcomes are based on the general inclusion of specific events/prescriptions (availability of information) and not on the agreement of content between the two datasets. Descriptive evaluations were performed with Microsoft SQL Server 2008 and Microsoft Excel 2010 (Microsoft Corporation, Redmond, WA, USA) software. All other statistical analyses were performed using Stata version 14.1 software (Stata Statistical Software: Release 14. StataCorp, College Station, TX, USA).

## Results

**Fig. 1:**
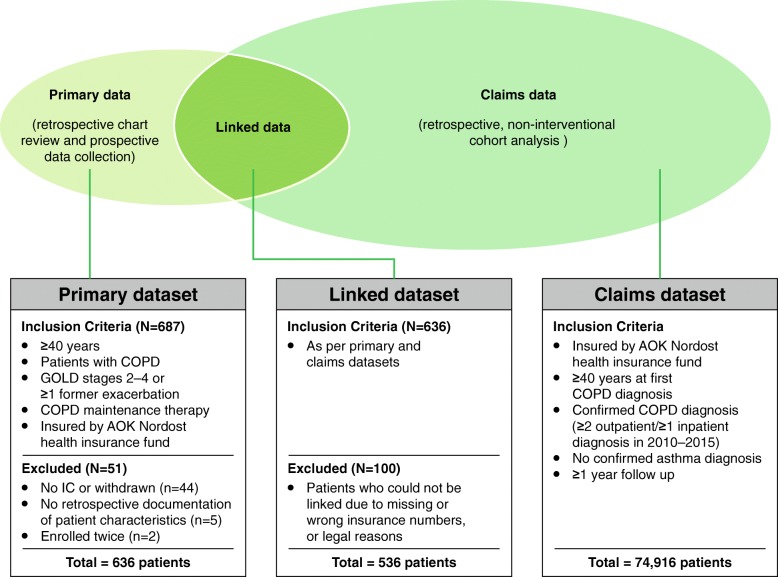
Inclusion criteria and sample sizes for each dataset. COPD, chronic obstructive pulmonary disease; GOLD, Global initiative for chronic Obstructive Lung Disease; IC, informed consent

The primary dataset included 636 patients with COPD who met all primary study inclusion criteria (mean [SD] age 68.1 [10.1] years; 38.1% female) and the claims dataset included 74,916 patients with COPD (mean [SD] age 70.9 [11.7] years; 46.0% female) (Fig. 1). Primary and claims data could be linked for 536 patients (mean [SD] age 68.0 [9.9] years; 36.4% female), who were included in the linked dataset. Overall, 100 patients from the primary dataset could not be linked due to incorrect or missing insurance numbers or legal reasons (e.g. patients were employees of AOK Nordost). Apart from gender, there were no obvious differences between linked and unlinked patients. Patient characteristics in all datasets are described in Table 1.

**Table 1:** Patient characteristics in the different datasets

	Primary dataset *N* = 636	Primary dataset patients not linked *N* = 100	Linked dataset *N* = 536		Claims dataset *N* = 74,916
	Based on primary data	Based on primary data	Based on primary data	Based on claims data	Based on claims data
Age, years					
Mean (SD)	68.1 (10.1)	68.6 (11.0)	68.0 (9.9)	68.5^a^ (9.9)	70.9 (11.7)
Median (IQR)	69 (15)	70 (16)	69 (15)	69 (14)	73 (18)
Female gender, n (%)	242 (38.1)	47 (47.0)	195 (36.4)	195 (36.4)	34,448 (46.0)
Smoking, n (%)					
Smoker	218 (34.3)	32 (32.0)	186 (34.7)	247 (46.1)	16,076 (21.5)
Former smoker	400 (62.9)	67 (67.0)	333 (62.1)		
Non-smoker	17 (2.7)	1 (1.0)	16 (3.0)		
Not-specified	1 (0.2)	0 (0.0)	1 (0.2)		
Comorbidities, n (%)^b^					
Hypertension	287 (45.1)	44 (44.0)	243 (45.3)	450 (84.0)	59,153 (79.0)
Diabetes (Type 1 or 2)	143 (22.5)	24 (24.0)	119 (22.2)	189 (35.3)	27,905 (37.2)
Depression	48 (7.6)	12 (12.0)	36 (6.7)	157 (29.3)	17,647 (23.6)
Osteoporosis	50 (7.9)	7 (7.0)	43 (8.0)	99 (18.5)	12,364 (16.5)
FEV_1,_ L^c^					
Mean (SD)	1.50 (0.6)	1.56 (0.7)	1.50 (0.6)	NA	NA
Median (IQR)	1.4 (0.8)	1.4 (0.9)	1.4 (0.8)		
% of predicted FEV_1_^d^					
Mean (SD)	55.6 (17.4)	57.2 (18.2)	55.3 (17.2)	NA	NA
Median (IQR)	57.0 (25.3)	60.0 (26.4)	56.0 (25.8)		

*COPD* chronic obstructive pulmonary disease, *FEV*_1_ forced expiratory volume in 1 s, *ICD*-10, International Classification of Disease, 10th Edition, *IQR* interquartile range, *SD* standard deviation

Primary dataset: all data reported for index date except comorbidities (any known to study physician). Claims dataset: all data reported for date of first COPD diagnosis except comorbidities (from January 2010 to date of first COPD diagnosis). Linked dataset: all data reported for linked dataset index date except comorbidities (primary: any known to study physician; claims: from January 2010 to linked dataset index date)

Smoking status was identified in the claims data using ICD-10 code F17. Comorbidities were selected based on those most commonly reported which could be directly compared between primary and claims data using ICD-10 codes: diabetes: E10/E11; depression: F32/F33; osteoporosis: M80-M82; hypertension: I10-I15

^a^In the claims data, only birth year was available. Therefore, age at linked dataset index date was calculated based on the assumption that all patients were born on July 1 of the respective year

^b^Values were calculated for all patients for whom data were available (primary sample/linked sample): diabetes: 621/518; depression: 611/515; osteoporosis: 561/477; hypertension: 600/512

^c^Values were calculated for all patients for whom data were available (primary sample: *n* = 620; linked sample: *n* = 527)

^d^Values were calculated for all patients for whom data were available (primary sample: *n* = 612; linked sample: *n* = 522)

### Patient characteristics

Gender information at index was identical across the primary and claims datasets for all (100%) patients in the linked dataset. Information on age was identical for 513 patients (95.7%), and different for 23 patients (4.3%; mean difference: 9.4 years). Of the 536 patients in the linked dataset, 186 (34.7%) were identified as current smokers in the primary dataset compared with 247 (46.1%) in the claims dataset. The mean (SD) trough FEV_1_ for patients in the linked dataset was 1.50 (0.6), based on primary data; no data on trough FEV_1_ were documented in the claims dataset.

#### Comorbidities

The comorbidities which could be directly compared between the primary and claims dataset using respective ICD-10 codes were hypertension, diabetes (type 1 and type 2), depression and osteoporosis. A substantially smaller proportion of patients in the linked dataset had these comorbidities documented in the primary data compared with claims data (Table 2; all *p* < 0.001). This was confirmed in the sensitivity analysis which considered comorbidities as defined by at least two outpatient diagnoses, or at least one inpatient diagnosis (hypertension: 45.7% vs 82.3%, diabetes: 22.2% vs 34.7%, depression: 6.7% vs 27.8% and osteoporosis: 8.0% vs 17.5% for the primary and claims data, respectively). The degree of agreement between primary and claims data with regards to hypertension, diabetes and depression was higher in patients included by GPs compared with those included by pneumologists (differences were non-significant for osteoporosis; Table 2). Kappa coefficients showed a weak to strong degree of concordance for diabetes (range k = 0.58–0.91) and osteoporosis (range k = 0.36–0.66), and no to weak concordance for depression (range k = 0.09–0.59) and hypertension (range k = 0.26–0.40), regardless of whether data were collected by GPs or pneumologists (Table 2) [12].

**Table 2:** Prevalence rates of observed comorbidities in patients in the linked dataset

	Linked dataset (*N* = 536): percentage of patients with selected comorbidities								
	Primary data collected by GPs or pneumologists^a^			Primary data collected by GPs			Primary data collected by pneumologists		
	N = 536			*N* = 69			*N* = 467		
Comorbidities	PD	CD	Agreement^a^	PD	CD	Agreement^a^	PD	CD	Agreement^a^
Diabetes (Type 1 or 2)	22.2%	35.3%	87.5% (κ = 0.63)	43.5%	42.0%	98.5% (κ = 0.91)	19.1%	34.3%	84.8% (κ = 0.58)
Depression	6.7%	29.3%	77.7% (κ = 0.15)	15.9%	26.1%	89.8% (κ = 0.59)	5.4%	29.8%	75.6% (κ = 0.09)
Osteoporosis	8.0%	18.5%	90.5% (κ = 0.43)	20.3%	23.2%	97.1% (κ = 0.66)	6.2%	17.8%	88.4% (κ = 0.36)
Hypertension	45.3%	84.0%	63.6% (κ = 0.28)	62.3%	87.0%	75.3% (κ = 0.40)	42.8%	83.5%	59.3% (κ = 0.26)

*CD* claims dataset, *GP* general practitioner, *PD* primary dataset

Significance tests for differences in the percentage of patients diagnosed by GPs and pneumologists in the two datasets: diabetes: *p* = 0.030; depression: *p* = 0.007; osteoporosis: *p* = 0.129; hypertension: *p* = 0.005

PD values represent the percentage of patients with a particular comorbidity reported in primary data. CD values represent the percentage of patients with a particular comorbidity reported in claims data. Agreement was calculated at a patient level in each of the subgroups (overall linked dataset, primary data collected by GPs, primary data collected by pneumologists) as follows: percent agreement = 100 * ([number of patients in the subgroup with the comorbidity reported in both primary and claims data] + [number of patients in the subgroup with the comorbidity not reported in either primary or claims data]) / (total number of patients in the subgroup)

^a^Includes Cohen’s kappa coefficient for agreement between comorbidities recorded in the primary and claims datasets

The sensitivity analysis which also included prescriptions for the definition of comorbidities was only performed for hypertension and diabetes, as these comorbidities are associated with well-defined medications. Of the patients who received a diagnosis of hypertension in the claims data but not in the primary data (*n* = 187), 82.9% had at least one prescription for antihypertensive drugs (ATC Code C02- or C07-). For patients diagnosed with diabetes in the claims dataset but not the primary dataset (*n* = 73; among them *n* = 71 with type 2 diabetes), 35.6% had at least one prescription for antidiabetic drugs (ATC Code A10-).

### Prescriptions

**Fig. 2:**
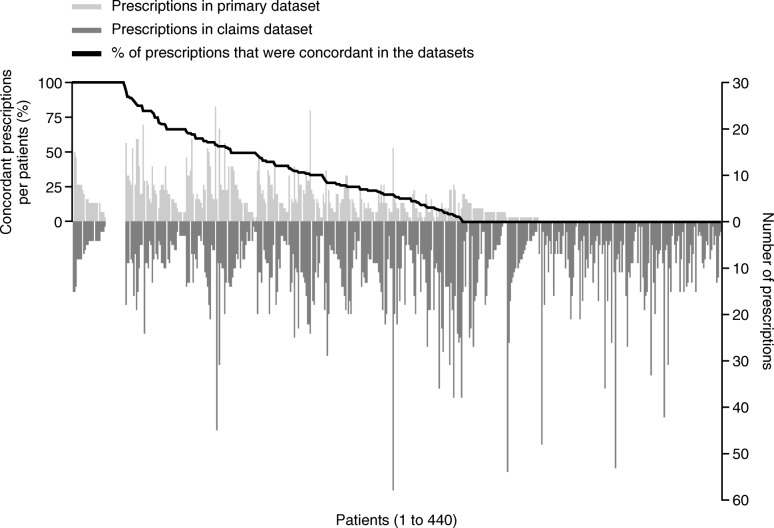
Agreement of observed COPD-related prescriptions between datasets. Data presented for the 440 patients in the linked dataset with complete documentation in the 12-month post-index period. COPD, chronic obstructive pulmonary disease

Of the 536 patients in the linked dataset, 96 patients had incomplete documentation for the 12-month post-index period in the primary dataset (at least one documentation at index, 6-month or 12-month missing), and were therefore excluded from the analysis of prescriptions. For the 440 patients from the linked dataset included in the analysis of prescriptions, the mean number of documented prescriptions per patient during the 12-month post-index period (overall and COPD maintenance therapies only) was smaller in the primary dataset compared with the claims dataset (overall: 3.7 vs 10.3 prescriptions per patient; COPD maintenance therapies: 2.7 vs 7.5 prescriptions per patient). Overall, 24.5% of prescriptions were found to be uniformly documented in both datasets, 8.6% were only documented in the primary dataset, and 66.9% were only documented in the claims dataset. When looking at COPD maintenance therapies only, 27.4% were documented in both datasets, 7.0% were documented only in the primary dataset, and 65.5% were documented only in the claims dataset. When evaluating the degree of agreement between primary and claims COPD prescription data for each patient, only 8.2% of patients showed 100% agreement of prescriptions between the two datasets, whereas no (0%) agreement was observed for 40.0% of patients (Fig. 2). There was no statistical difference in the degree of agreement between primary and claims data for COPD prescriptions documented by a pneumologist (agreement: 29.8%) compared with data documented by a GP (agreement: 24.0%; *p* = 0.081).

### Exacerbations

**Fig. 3:**
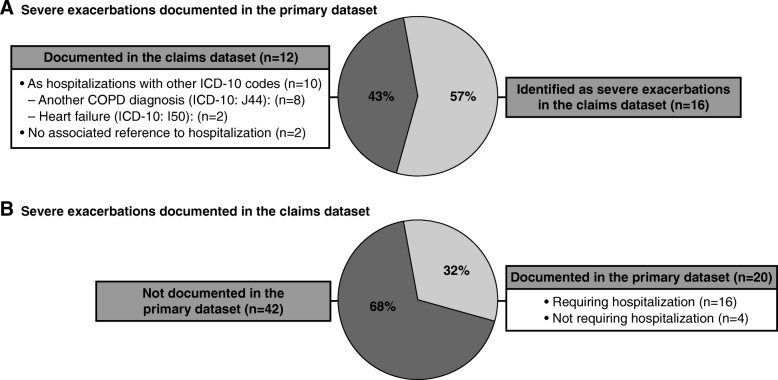
Agreement of severe exacerbations between datasets. Data presented for the 440 patients in the linked dataset with complete documentation in the 12-month post-index period. Part A presents severe exacerbations documented in the primary dataset. Part B presents severe exacerbations documented in the claims dataset. COPD, chronic obstructive pulmonary disease; ICD-10, International Classification of Diseases, 10th revision; n, number of severe exacerbations

Documented exacerbation events were compared between the primary and claims datasets for 440 patients in the linked dataset with complete primary data documentation during the 12-month post-index period (Table 3). Based on primary data, 92 (20.9%) patients experienced at least one moderate or severe exacerbation, with a mean of 1.4 exacerbations during the 12-month post-index period (Table 3). Respective numbers based on claims data were 128 (29.1%) patients with a mean of 3.0 exacerbations. Based on primary data, 26 (5.9%) patients in the linked dataset experienced at least one severe exacerbation (Table 3; 28 severe exacerbations in total); 16 of these 28 exacerbations (57.1%) were identified as severe exacerbations in the claims dataset (Fig. 3a). A detailed analysis of claims data for the remaining 12 hospitalizations showed that 10 were documented as hospitalizations with other ICD-10 codes (hospitalizations with another COPD diagnosis [ICD-10: J44]: *n* = 8; heart failure [ICD-10: I50]: *n* = 2), while two had no associated reference to hospitalization in the claims (Fig. 3a). Based on claims data, 45 (10.2%) patients experienced at least one severe exacerbation (Table 3; 62 severe exacerbations in total); 20 of these 62 exacerbations (32.3%) were documented in the primary dataset (requiring hospitalization: *n* = 16; not requiring hospitalization: *n* = 4; Fig. 3b).

**Table 3:** Documented exacerbation events in the linked dataset

	Linked dataset (*N* = 440)	
	Based on primary data	Based on claims data
Moderate and severe exacerbations		
Number of exacerbations during the 12-month post-index period, mean (SD)	1.4 (0.8)	3.0 (2.1)
Patients with ≥ 1 exacerbation, n (%)	92 (20.9)	128 (29.1)
Severe exacerbations		
Patients with ≥ 1 severe exacerbation, n (%)	26 (5.9)	45 (10.2)

Data presented for the 440 patients in the linked dataset with complete documentation in the 12-month post-index period

Severe exacerbations: exacerbations requiring hospitalization (primary dataset), hospitalizations associated with an exacerbation ICD-10 code (claims dataset); *n* number of patients

**Fig. 4:**
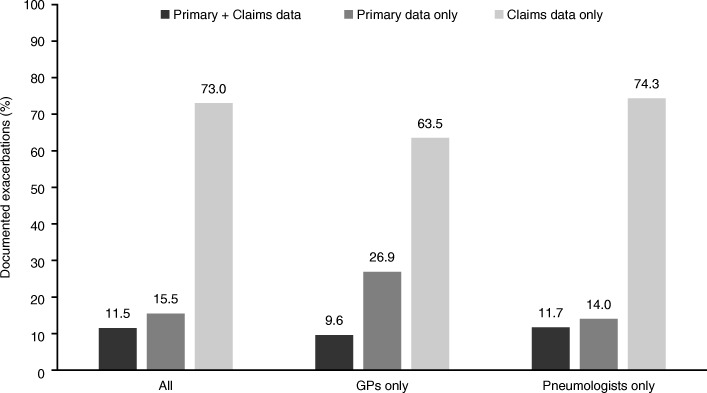
Agreement of exacerbations documented in the datasets by healthcare professionals. Data presented for the 440 patients in the linked dataset with complete documentation in the 12-month post-index period. GP, general practitioner

Overall, 11.5% of exacerbations were documented in both primary and claims data, 15.5% were documented only in primary data, and 73.0% were documented only in claims data (Fig. 4). The agreement between the primary and claims datasets was 11.7% for exacerbations documented by pneumologists (14.0% and 74.3% of exacerbations solely documented in primary and claims data, respectively), and 9.6% for exacerbations documented by GPs (26.9% and 63.5% of exacerbations solely documented in primary and claims data, respectively; *p* = 0.053 for the comparison with agreement for exacerbations documented by pneumologist; Fig. 4).

## Discussion

This study evaluated agreement between primary and secondary data collection (prospective observational data/retrospective chart review and retrospective claims data, respectively). We found discrepancies between primary data and claims data for linked patients for most assessed variables including comorbidities, drug prescriptions, and exacerbations, with a lower number of comorbid patients, COPD therapy prescriptions and exacerbations reported in primary versus claims data.

Agreement between primary and claims datasets with regards to sociodemographic data was very good. The gender information was identical between datasets and the information on age differed only in a small number of patients (4.3%, mean difference 9.4 years). This difference could be associated with recording errors or missing data in the primary or claims datasets; however, as the mean age for linked patients was similar between the primary and claims data, it is unlikely that this reflects any major discrepancies between the datasets.

Kappa values for the prevalence rates of observed comorbidities indicated no to strong agreement between the primary and claims datasets, based on previous interpretations that indicated that values ≤0.20 indicate no agreement, 0.21–< 0.40 minimal agreement, 0.40–< 0.60 weak agreement. 0.60–< 0.80 moderate agreement, 0.80–0.90 strong agreement and > 0.90 almost perfect agreement [12]. We initially expected to observe a higher percentage of comorbid patients in the primary data compared with the claims data as physicians were asked to document all comorbidities that they were aware of in the primary data collection whereas the claims data analysis only considered comorbidities documented by a respective ICD-10 code between January 1, 2010 and the patient’s linked dataset index date. Instead, the percentage of comorbid patients was higher using claims data compared with primary data; this difference was even more pronounced in patients included by pneumologists in the primary dataset, as indicated by the lower kappa values within this subset. Specific limitations of the data sources may explain these deviations. One limitation of using primary data collection to document comorbidities is that study physicians, particularly disease specialists such as pneumologists, may not be aware of every comorbidity a patient suffers from. This may explain why we found that GPs generally documented more comorbidities compared with pneumologists in the primary data, as GPs consider the overall health of the patient, which is reflected in the better agreement between datasets for comorbidity data collected by GPs versus those collected by pneumologists, as indicated by the higher kappa values. On the other hand, claims data collection is also limited as comorbidities may be more frequently reported owing to the prescription behavior of German physicians being evaluated by payers based on documented background diagnoses. However, since most patients who were classified as comorbid in claims data only were also given respective drug therapies, this limitation can only partially explain the differences.

This study also showed that only approximately a quarter (24.5%) of prescriptions for COPD therapies were recorded in both datasets, with a lower number of prescriptions generally observed in primary data. These findings could be due to incomplete data collection in the primary dataset. Incomplete data collection may arise when physicians other than the primary study physician prescribe COPD medications, thereby leading to lower reporting of prescription data in primary data collection in the absence of electronic medical records. Additionally, prescriptions found in primary data but not in claims data may be explained by non-filled receipts, indicating patients’ primary non-adherence. Therefore, study conclusions drawn from the analysis of primary data only could miss a substantial number of patient prescriptions, while studies based on claims data only cannot capture patient primary non-adherence, highlighting the potential benefits of using a linked dataset to more fully describe treatments received by study patients.

We also observed substantial differences in documented exacerbations (moderate and severe) between the two datasets. The higher number of documented exacerbations in the claims data may be due to specific features of the German outpatient coding system. In this system, physicians can “keep” a diagnosis in their practice software and re-document it at the next visit; on the other hand, the system only allows researchers to record a specific ICD-10 code once per quarter for every attending physician, which can potentially lead to lower reporting as the maximum number of events reported by quarter is one. As kappa coefficients were not calculated for the exacerbation results, this potential under-reporting should be stressed. However, specifics of the coding system cannot explain the substantial differences in the number of severe exacerbations (leading to hospitalizations) documented in the datasets. The lower number of moderate or severe exacerbations observed in the primary dataset compared with the claims dataset could be due to study physicians not being fully appraised of the patient’s pathways, treatments and hospitalizations that they did not themselves initiate or steer. In this regard, it is interesting to note the slightly higher agreement between datasets for exacerbations documented by pneumologists compared with those documented by GPs, possibly due to the higher awareness of disease-specific events among specialists. Overall, our findings indicate that data linkage between primary and secondary data sources could increase the validity of data when describing exacerbation events in patients with COPD.

Results from this study highlight some benefits and limitations of the data sources considered. One of the main advantages of primary data collection is that it allows the selection of data that are of direct interest to the researcher; however, it may be limited by a risk of lower reporting and incomplete data collection. On the other hand, claims data collection can give access to a greater amount of data, but may be limited by the selection of variables available [13]. The limitations of primary data collection are particularly apparent for information related to patient treatment provided by physicians or institutions that did not participate in the study. For example, the risk of lower reporting is likely to be higher for variables that can be measured and influenced by multiple healthcare professionals (e.g., GPs, different specialists and hospitals) such as drug treatment and exacerbations, whereas disease-related variables that require specific equipment or knowledge for physician assessment are less likely to be affected. This is also relevant to the interpretation of data stemming from registries, which are typically designed as primary prospective observational studies with specialists documenting registry data. Furthermore, patients who agree to participate in prospective data collection may not be representative of the wider patient population, and in this regard claims databases may provide higher external validity compared with primary datasets. However, claims data may themselves be limited by the availability of recorded information or the risk of higher reporting of specific items that are associated with positive reimbursement decisions (as noted above). For example, in German claims data, only data associated with the reimbursement of services (hospitalizations, prescriptions and outpatient treatments) are generally available [14], whereas disease-specific laboratory values and COPD-specific outcomes such as lung function, COPD symptoms or GOLD group are not captured. Therefore, using COPD as an example, a claims data-based study may describe patient characteristics, prescribed medications and outpatient/inpatient treatments, but may be unable to report important disease characteristics such as lung function, COPD Assessment Test (CAT) score, modified Medical Research Council (mMRC) score or laboratory values, unless these are available in the claims database considered.

This analysis showed that data linkage of primary and claims datasets can lead to data enrichment, in certain situations such as the analysis of drug prescriptions and the reporting of exacerbation events. For example, in this study, the claims dataset captured a higher number of recorded prescriptions compared with the primary dataset, as illustrated in Fig. 2. The primary dataset contains data recorded by only the study physicians, whereas the claims dataset also gives access to data recorded by physicians other than the primary study physician, thereby providing additional details which would not be captured in the primary data. The results show that linking primary and claims data for the recording of prescriptions can yield a more complete description of the data. The value of data linkage has also been demonstrated in other disease areas, for example, in a comparison between cancer registries and GP electronic health records in England [15]. Other studies have also highlighted the added value of data linkage with regards to improving disease identification [10]. However, data linkage could potentially introduce selection bias [10]. Further analysis of data from our study may provide insight on whether data linkage introduced such a bias in this example.

This study is not without limitations. Our conclusions are based on German data and therefore influenced by data capture methodology specific to Germany; other databases in other countries may identify additional benefits and limitations not covered by our observations. Additionally, the German healthcare system is characterized by a widespread network of outpatient specialists operating outside of hospital care, increasing the probability that patients visit specialists independently, unbeknown to their regular physicians (GPs or other specialists). Furthermore, inclusion of patients was based on slightly different criteria in each dataset. The main reasons for this were the need to also address further research questions such as general prevalence of COPD in Germany (not presented here), and unavailability of applied criteria in one or the other dataset. Nevertheless, as the content of this publication focuses on linked patients only, we do not expect this to have any impact on the presented analysis. In addition, not all variables collected in the primary data were documented in the claims dataset, for example spirometry measures, CAT score, mMRC score, and sociodemographic characteristics such as educational level and professional activity. Agreement between datasets with regards to these variables could therefore not be assessed.

Moreover, the observation periods for documenting comorbidities were different in the primary (any comorbidities known to the physician at linked dataset index date) versus the claims datasets (January 2010 to linked dataset index date). However, as the comorbidities analyzed here were chronic diseases, this discrepancy in reporting periods may not have overly influenced the differences observed between datasets. The study did not consider potential linking errors, including potential errors in recording of patients’ insurance numbers, which could have contributed to the differences observed between the datasets. Finally, the inherent weaknesses associated with primary data collection may have contributed to the observed lower reporting of comorbidities, prescriptions and exacerbations relative to claims data collection, despite the primary study being performed in accordance with all known guidelines for observational research, and including on-site visits and extensive data validity control.

In conclusion, this study highlights discrepancies between primary and claims data collection capture for this population of German patients with COPD. Primary data collection may be appropriate for studies primarily assessing information that is completely available at one study site (e.g., diagnostic data, especially when required equipment is only available at these sites) or when the risk of patient and study site selection bias is minimized by random or consecutive sampling. An analysis based on claims data may be effective in observational COPD research in situations where only the variables that are well covered in the claims data are of interest (such as costs, hospitalizations and outpatient prescriptions). In other situations, linking primary and secondary data sources for the same patient population could enrich data and may be a preferred choice to fully describe COPD endpoints.

## Additional file

Additional file 1: Supplementary methods, figure and tables. (DOCX 71 kb)

## Abbreviations

ATC: Anatomical Therapeutic Chemical classification scale
CD: Claims dataset
COPD: Chronic obstructive pulmonary disease
FEV_1_: Forced expiratory volume in 1 s
GOLD: Global initiative for chronic Obstructive Lung Disease
GP: General practitioner
ICD-10: International Classification of Diseases, 10th revision
ICS: Inhaled corticosteroids
IQR: Interquartile range
LABA: Long-acting β_2_-agonists
LAMA: Long-acting muscarinic antagonists
PD: Primary dataset
PDE-4: Phosphodiesterase type 4 inhibitor
RWE: Real-world evidence
SABA: Short-acting β_2_-agonists
SAMA: Short-acting antimuscarinic antagonists
SD: Standard deviation
