# Supplementary material for: Primary data, claims data, and linked data in observational research: the case of COPD in Germany
Source: Respir Res. 2018 Aug 30;19:161. doi: 10.1186/s12931-018-0865-1 (PMC6117888; doi:10.1186/s12931-018-0865-1)
Supplement: Supplementary file 1 — Supplementary methods, figure and tables. (DOCX 71 kb) [file 12931_2018_865_MOESM1_ESM.docx]

**Supplementary Materials**

**Supplementary Methods**

*Patient-reported outcomes*

Physicians handed a standardized questionnaire to their patients at index as well as approximately
6 and 12 months after study inclusion at study site visits. Two structured patient telephone interviews were conducted by trained interviewers at approximately 9 months after index and at the end of the 12-month post-index observational period. Supplementary Figure 1 shows the structure of the different data collection methods and collection periods for primary data collection.

**Additional file 1: Figure S1: Primary data collection process**


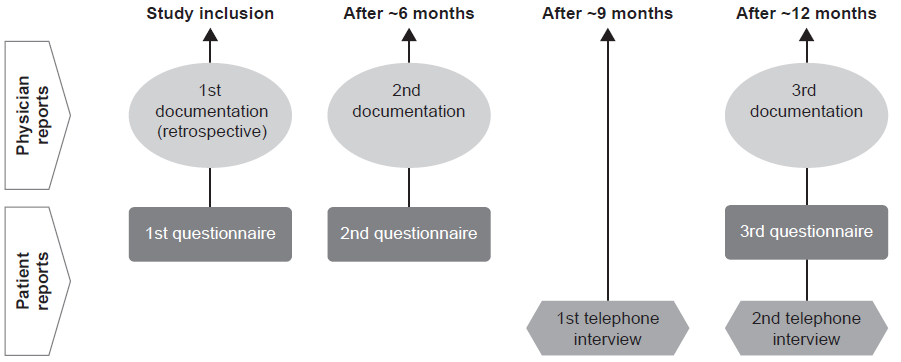


**Additional file 1: Table S1: Available data in the primary dataset**

|  |  |  |  |
| --- | --- | --- | --- |
|  | **Data documented at inclusion visit** | **Data documented at 6-month visit** | **Data documented at 12-month visit** |
|  |  |  |  |
| Age | X |  |  |
| Gender | X |  |  |
| Life circumstances | X |  |  |
| Education | X |  |  |
| Working activities | X |  |  |
| Sports activities | X |  |  |
| Height | X |  |  |
| Weight | X |  |  |
| Smoking status | X |  |  |
| Years of smoking and number of cigarettes/day | X |  |  |
| Comorbidities* | X |  |  |
| COPD phenotype | X |  |  |
| COPD-related therapy recommendations | X |  |  |
| Vaccinations* | X |  |  |
| COPD-related bullectomy* | X |  |  |
| Exacerbations (including date, severity, and treatment)* | X | X | X |
| COPD-related hospitalizations | X | X | X |
| Spirometry measures* | X | X | X |
| Prescribed COPD medication and recommended dose* | X | X | X |
| Number of long-term COPD medications | X | X | X |
| Changes in long-term COPD therapy and reasons |  | X | X |
| COPD therapy breaks and reasons |  | X | X |

*Data were collected retrospectively (24 months or more) and prospectively (12 months). COPD, chronic obstructive pulmonary disease.

**Additional file 1: Table S2: COPD-related medication classes**

| **Medication class** | **Agents** | **ATC codes** |
| --- | --- | --- |
| SABA | Salbutamol | R03AC02 |
|  | Terbutaline | R03AC03 |
|  | Fenoterol | R03AC04 |
| LABA | Salmeterol | R03AC12 |
|  | Formoterol | R03AC13 |
|  | Indacaterol | R03AC18 |
|  | Olodaterol | R03AC19 |
|  | Bambuterol | R03CC12 |
|  | Clenbuterol | R03CC13 |
| LABA-combi | Clenbuterol combinations | R03CC63 |
| SABA-combi | Fenoterol and sodium cromoglicate | R03AK03 |
|  | Salbutamol and sodium cromoglicate | R03AK04 |
|  | Reproterol and sodium cromoglicate | R03AK05 |
| ICS+LABA | Salmeterol and fluticasone | R03AK06; R03AK61 |
|  | Formoterol and budesonide | R03AK07; R03AK28; R03AK72 |
|  | Formoterol and beclometasone | R03AK08; R03AK27; R03AK71 |
|  | Vilanterol and fluticasone furoate | R03AK10 |
|  | Formoterol and fluticasone | R03AK11 |
| SABA+SAMA | Fenoterol and ipratropium bromide | R03AL01 |
|  | Salbutamol and ipratropium bromide | R03AL02 |
| LABA+LAMA | Vilanterol and umeclidinium bromide | R03AL03 |
|  | Indacaterol and glycopyrronium bromide | R03AL04 |
|  | Formoterol and aclidinium bromide | R03AL05 |
|  | Olodaterol and tiotropium bromide | R03AL06 |
| ICS | Beclometasone | R03BA01 |
|  | Budesonide | R03BA02 |
|  | Fluticasone | R03BA05 |
| SAMA | Ipratropium bromide | R03BB01 |
| LAMA | Tiotropium bromide | R03BB04 |
|  | Aclidinium bromide | R03BB05 |
|  | Glycopyrronium bromide | R03BB06 |
| Methylxanthine | Theophylline | R03DA04 |
|  | Aminophylline | R03DA05 |
| PDE-4 | Roflumilast | R03DX07 |

COPD, chronic obstructive pulmonary disease; ICS, inhaled corticosteroids; LABA, long-acting β_2_-agonists; LAMA, long-acting muscarinic antagonists; PDE-4, phosphodiesterase type 4 inhibitor; SABA, short-acting β_2_ agonists; SAMA, short-acting muscarinic antagonist.
